# Influence of Anthropometrics on Step-Rate Thresholds for Moderate and Vigorous Physical Activity in Older Adults: Scientific Modeling Study

**DOI:** 10.2196/12363

**Published:** 2018-12-13

**Authors:** Myles William O'Brien, Matthew Jordan Kivell, William Robert Wojcik, Ghislain Richard D'Entremont, Derek Stephen Kimmerly, Jonathon Richard Fowles

**Affiliations:** 1 Centre of Lifestyle Studies School of Kinesiology Acadia University Wolfville, NS Canada; 2 Division of Kinesiology Dalhousie University Halifax, NS Canada

**Keywords:** aging, walking, public health, cadence, physical activity intensity

## Abstract

**Background:**

Adults and older adults are recommended to engage in 150 minutes of moderate (MPA) to vigorous (VPA) aerobic physical activity (MVPA) per week, with the heuristic message of 3000 steps in 30 minutes (100 steps per minute [spm]). However, this message is based on adult populations, with a paucity of research on step-rate thresholds that correspond to absolute MVPA (moderate=3 metabolic equivalents [METs], vigorous=6 METs) and relative MVPA (moderate=40% estimated MET_max_, vigorous=60% estimated MET_max_) in older persons, who have lower stride lengths and a lower exercise capacity. Also, there is a need to consider the influence of anthropometric differences when quantifying the relationship between step rate and intensity-related physical activity.

**Objective:**

This study assessed absolute and relative MVPA step-rate thresholds and anthropometric factors (ie, height, leg length, and body mass index [BMI]) in older adults.

**Methods:**

Nineteen older adults (7 females; age 69 years, SD 2, BMI 26 kg/m^2^, SD 4) completed a staged treadmill walking protocol: six minutes at 2.4, 3.2, 4.0, 5.6, and 6.4 km/h. Steps were manually counted and volume rate of oxygen consumed (VO_2_) was measured via indirect calorimetry. Aerobic fitness was estimated via the submaximal single-stage treadmill protocol.

**Results:**

When BMI was considered, mixed effects modeling revealed absolute and relative MPA step-rate thresholds of 108 spm and 117 spm, respectively. Absolute and relative VPA corresponded to step rates of 135 spm and 132 spm, respectively. Neither height nor leg length improved the ability of the model to predict stepping cadence from METs.

**Conclusions:**

In general, older adults need to walk faster than 100 spm (ie, approximately 110 spm) to reach MPA and in excess of approximately 130 spm to achieve VPA, depending on BMI status. Health care professionals and researchers should adjust cadence-based recommendations for differences in BMI in their older patients and consider using relative intensity to most appropriately tailor their physical activity recommendations.

## Introduction

Leading a physically active lifestyle is associated with numerous health benefits among older adults that include improved cardiovascular and metabolic health and a reduction in all-cause mortality [1-3]. When objectively measured, only 12% of Canadian older persons achieve the national physical activity guidelines that recommend 150 minutes of moderate-to-vigorous aerobic physical activity (MVPA) per week [4]. Older adults report walking as the most common form of physical activity [5], with research showing that MVPA is attainable in this population [6].

Initially, 3000 steps in 30 minutes (or 100 steps per minute [spm]) has been proposed as a heuristic cadence-based recommendation to achieve moderate-intensity physical activity (MPA) (3 metabolic equivalents [METs]) in adults [7]. More recent studies have validated this public message [8-10]; however, most research has been conducted in samples of young adults with few studies investigating this relationship in older adults [11,12]. Older adults experience physiological and biomechanical changes that decrease their exercise capacity and stride length [13,14]. These age-related processes limit the generalizability of cadence-based MVPA thresholds that are based on young adult samples.

Many studies have quantified MVPA in terms of absolute intensity, where MPA and vigorous-intensity physical activity (VPA) are defined as 3 METs and 6 METs, respectively. However, the Canadian Society of Exercise Physiology (CSEP) [15] and American College of Sport Medicine [16] recommend using individualized intensities for exercise prescription, where MPA is 40% of maximal aerobic fitness (VO_2max_) and VPA is 60% of VO_2max_. Relative metabolic intensities may have greater applicability to older adults who, on average, are less aerobically fit and benefit from individualized exercise prescriptions [17-19]**.**

As highlighted in a recent narrative review on the topic [20], there are only two studies that have examined MPA step-rate thresholds in older populations, and they report conflicting findings. Serrano et al [12] observed that 40% VO_2reserve_ (mean 3.3 METs, SD 0.8) was associated with approximately 115 spm and that body weight and self-selected walking cadence best predicted (*R*^2^=0.34) the cadence required to reach relative MPA. However, Peacock et al [11] observed that absolute MPA step-rate thresholds were lower in older adults than their previously published sample of young adults [21] when matched for height (at 170 cm: young=104 spm; older=91 spm) and that METs were best predicted (*R*^2^=0.50) when step rate, height, and age were predictor variables. Interestingly, both studies analyze the cadence–intensity relationship using linear methods, whereas this relationship has been shown to be curvilinear [8,9,22-24]. Despite the inconsistency in the existing literature, both studies clearly demonstrate that anthropometrics (ie, height or body weight) alter MVPA step-rate thresholds in that shorter and/or lighter individuals generally need to take more steps to reach the same intensity as their taller and/or heavier counterparts.

Given the disagreement in the current literature regarding appropriate MPA step cadences for older persons as well as the lack of consideration for VPA, there is a need to investigate step-rate recommendations for both absolute and relative MPA and VPA in an aged population. Therefore, the purpose of this study was to use mathematical modeling with anthropometric factors as predictor variables to calculate older adults’ individualized step rate. Using these models, we will determine step-rate thresholds that define both absolute and relative MPA and VPA in our sample of older adults.

## Methods

### Demographics

Nineteen older adults (7 females) aged mean 68.8 (SD 2.3; 65-74) years volunteered to participate in this study. All participants were initially screened for age (over 65 years) and cleared for MVPA using the Physical Activity Readiness Questionnaire Plus (PAR-Q+) [25]. All participants completed a CSEP Physical Activity and Sedentary Behavior Questionnaire (PASB-Q), a valid and reliable measure of weekly MVPA [26]. The study was approved by the Research Ethics Board at Acadia University (REB#15-20), and all subjects provided written informed consent before participating. All participants were recruited via a community-wide email and by word of mouth in Wolfville, Nova Scotia, from May 2015 to September 2015.

### Anthropometrics

Height and weight were measured without shoes using a calibrated stadiometer and scale (Health-O-Meter, Sunbeam Products Inc) to the nearest 0.5 cm and 0.1 kg, respectively. Leg length was measured with participants in the seated position using a tape measure (cm) as the distance from the greater trochanter to the floor without footwear.

### Aerobic Fitness

Aerobic fitness was estimated using the validated Ebbeling walking treadmill protocol [27]. The Ebbeling consists of two 4-minute walking stages. The first stage is designed to reach a speed that elicits approximately 60% of the participants’ estimated heart rate maximum (ie, 220–age), and the second stage involves increasing the treadmill grade by 5%. Treadmill speed and steady-state heart rate are used to estimate VO_2max_ [28]. A submaximal test was chosen over a maximal test for safety reasons and to minimally influence the subsequent walking assessment. Furthermore, the time frame of this particular test (8 minutes) corresponds to the time restraints experienced by qualified exercise professionals to counsel, assess patients’ physical fitness, and produce an optimal exercise program. Following the submaximal aerobic test, a resting period of 20 to 30 minutes was allotted to ensure participants returned to a rested state.

### Treadmill Protocol

The study design and protocol were adapted from our previous investigation in young adults [29]. Prior to testing, the metabolic cart (TrueOne 2400, Parvo Medics) was calibrated using nitrogen and 2 primary standard gas mixtures to an error of 0.01%. The pneumotachometer was calibrated using a 3-L syringe that delivered fixed volumes at different flow rates. Volume calibration was verified to a value less than 0.1 L. Heart rate was monitored using a telemetry transmitter attached across the sternum (T31, Polar Electro). Participants were familiarized with the Borg scale and asked to estimate their rating of perceived exertion (RPE) on a scale of 6 to 20 [30]. Participants were fitted with a headpiece, a 2-way nonrebreathing valve (Hans-Rudolph Inc), a noseclip, and a mouthpiece.

Participants performed up to five 6-minute walking bouts on a calibrated, level treadmill at 2.4, 3.2, 4.0, 5.6, and 6.4 km/h (1.5, 2.0, 2.5, 3.5, and 4.0 mph, respectively). Each bout was separated by a 4-minute standing rest period to ensure minimal drift of metabolism between stages. The order of treadmill bouts was progressive because of concerns that some participants would be unable to walk at the higher speeds due to limitations in fitness. Considering the relationship between step rate and metabolic activity is likely altered at jogging and running paces in comparison to walking, only walking was permitted.

Steps were manually counted by two researchers during minutes 2 to 3 and 4 to 5 of each stage in order to obtain the gold standard step counts per minute for each stage. A video camera filmed the feet of the participant in case the researchers recorded greater than 1 step difference during a stage. The steps counted during minutes 2 to 3 and 4 to 5 of each stage were averaged and multiplied by a factor of 6 to determine the number of steps for each 6-minute stage. Of relevance, the steps counted during minutes 2 to 3 and 4 to 5 were always within 2 spm. The test was terminated by completing the protocol of all 5 stages, volitional fatigue, or if the participant reached 85% of their estimated heart rate maximum or RPE was greater than 17 [28]. An appropriate cooldown was administered by the researcher while monitoring the participant’s heart rate recovery.

Steady-state volume rate of oxygen consumed (VO_2_) for each 6-minute walking bout was obtained using indirect calorimetry, where steady state was defined as a heart rate change of less than 5 beats per minute, consistent with previous research [10]. VO_2_ and heart rate data were recorded at 15-second intervals for the duration of the protocol. Steady-state VO_2_ for each participant was recorded as an average of the last 4 minutes of each bout to limit the variability introduced by oxygen kinetics at the onset of each protocol stage.

### Data and Statistical Analysis

Statistics were completed in R version 3.4.1 (R Foundation for Statistical Computing) and SPSS Statistics for Mac version 23.0 (IBM Corp). Descriptive statistics are presented in the text as mean and standard deviation or proportion (%). Statistical significance was accepted as *P*<.05.

Participants’ relative estimated VO_2max_ was divided by 3.5 mL/kg/min to calculate their maximum METs. For absolute intensity, MPA and VPA were classified as 3.00 to 5.99 METs and >6.00 METs, respectively. For relative intensity, MPA and VPA were classified as 40% to 59% MET_max_ and >60% MET_max_, respectively. For each stage, METs were calculated by dividing steady-state VO_2_ by 3.5 mL/kg/min. Stepping rate was computed as the average between manually counted steps during minutes 2 to 3 and 4 to 5 of each stage. Step length (meters per step) was determined by dividing treadmill speed (meters per minute) by step rate.

Multiple regression, mixed effects models, and receiver operating characteristic (ROC) curve analyses were used to differentiate step-rate cut points for both MPA and VPA. The multiple regression approach was used to develop an equation that predicts step rate using metabolic activity and a combination of body mass index (BMI), height, and/or leg length, consistent with previous research [9,24]. However, multiple data points from each individual were used in the current analysis, thus violating the assumption of data independence. Therefore, mixed effects modeling was used to overcome this limitation by incorporating random intercepts to account for the data-dependence structure. ROC curves were used to evaluate optimal step-rate cut points that resulted in the highest sensitivity (true positives) and specificity (true negatives) for intensity-related physical activity as derived via Youden’s index. For ROC area under the curve (AUC) analysis, below 0.70 was considered poor, 0.70 to 0.80 considered fair, 0.80 to 0.90 considered good, and 0.90 to 1.00 considered excellent [31].

Subject-level plots of step rates as a function of METs indicated that the relationship between these variables was curvilinear. The model presented by Beets and colleagues [9], which used METs, METs^2^, BMI, leg length, and BMI×METs as predictors of step rate, was used as the starting model. Parameters were added or removed (eg, height, body weight, METs^3^) according to relative goodness of fit and model complexity and based on *a priori* knowledge regarding the relationship among the variables of interest and the objective of this study. Of the candidate models, relative Akaike information criterion (AIC) scores (a model comparison measure) were used to determine which among them were most probable to minimize the information loss (ie, which model was closer to the true model or the data-generating model). AIC comparisons were used to identify which models are best at trading off bias versus variance among the fitted model parameters [32]. As such, AIC comparisons were used to identify which models were expected to maximize predictive accuracy. Model diagnostics were run for the best mixed model for each height, body weight, BMI, and/or leg length as predictors. The inclusion of height, leg length, or body weight as predictor variables did not improve the predictive capabilities of the model, whereas BMI alone drastically increased the ability of model to predict step rates from metabolic activity. Assumptions of normality, homoscedasticity, and independence were assessed via residual plots. The relationship among predictors was assessed via scatter plots.

## Results

### Participants

On average, participants self-reported 234 (SD 157) minutes of MVPA per week. Most participants (13/19) reported at least one yes to a question on the PAR-Q+. The mean (SD; range) values of leg length, height, and BMI were 96.2 (SD 7.6; 82-108) cm, 169.8 (SD 7.3; 160-187) cm, 26.3 (SD 3.5; 20.6-31.2) kg/m^2^, respectively, with participants being classified as healthy BMI (18.6-24.9 kg/m^2^; 6/19) overweight (25.0-29.9 kg/m^2^; 9/19), or obese class one (30.0-34.9 kg/m^2^; 4/19). Median values for leg length, height, and BMI were 95.0 cm, 168 cm, and 26.2 kg/m^2^, respectively. The average estimated VO_2max_ of the sample was 31.0 ml/kg/min (SD 2.9; 25.7-35.7). Participants were classified as fair (7/19), good (7/19) or very good (5/19).

As demonstrated in Table 1, all outcome variables progressively increased with faster walking speeds. Subject relative moderate METs and relative vigorous METs were 3.5 (SD 0.3) METs (median 3.5 METs) and 5.3 (SD 0.5) METs (median 5.3 METs), respectively.

### Multiple Regression

The multiple regression model generated to predict MVPA step rates from METs and BMI is presented in Table 2. Step rates were accurately predicted when BMI was a predictor variable (*R*^2^=0.774; *P*<.001). Predicted absolute and relative intensity-related physical activity step-rate thresholds using the median values for BMI (26.2 kg/m^2^) are presented in Table 3.

**Table 1:** Description of measured variables at each treadmill speed.

Stage (speed)	Value, n	Step rate (spm^a^), mean (SD)	METs^b^, mean (SD)	VO_2_^c^ (mL/kg/min), mean (SD)	Step length (meters), mean (SD)	Heart rate (bpm^d^), mean (SD)	RPE^e^ (6-20), mean (SD)
Stage 1 (2.4 km/h)	19	91 (9)	2.4 (0.2)	8.4 (0.8)	0.45 (0.05)	83 (10)	8.5 (1.0)
Stage 2 (3.2 km/h)	19	104 (8)	2.7 (0.3)	9.5 (0.9)	0.52 (0.04)	85 (11)	9.5 (1.1)
Stage 3 (4.0 km/h)	19	110 (7)	3.1 (0.2)	10.7 (0.8)	0.61 (0.04)	88 (11)	10.7 (1.5)
Stage 4 (5.6 km/h)	18	125 (6)	4.4 (0.5)	15.2 (1.9)	0.75 (0.03)	105 (12)	12.2 (1.8)
Stage 5 (6.4 km/h)	14	133 (6)	5.7 (0.6)	19.9 (2.0)	0.81 (0.04)	117 (11)	13.5 (2.4)

^a^spm: steps per minute.

^b^METs: metabolic equivalents.

^c^VO_2_: volume rate of oxygen consumed (mL/kg/min).

^d^bpm: beats per minute.

^e^RPE: rating of perceived exertion (6-20).

**Table 2:** Multiple regression and mixed effects models to predicted step rate from metabolic equivalents.

Analysis	Model	Adjusted R^2^ (*P* value)
Multiple regression	–50.768 + (71.707×METs^a^) – (9.650×METs^2^) + (0.543×METs^3^) + (1.775×BMI^b^) – (0.394×BMI×METs)	0.774 (<.001)
Mixed methods^c^	–84.321 + (91.209×METs) – (12.968×METs^2^) + (0.772×METs^3^) + (2.211×BMI) – (0.549×BMI×METs)	—^d^

^a^METs: metabolic equivalents.

^b^BMI: body mass index (kg/m^2^).

^c^Recommended model to predict step rate.

^d^An R^2^ value is presented for the multiple regression but not the mixed method model.

**Table 3:** Minimum step rates for both absolute and relative moderate and vigorous intensity walking as established using multiple regression, mixed effects model, and receiver operating characteristic curve analyses.

Analysis	Intensity-related physical activity step rates			
	Absolute MPA^a^ (3 METs^b^)	Absolute VPA^c^ (6 METs)	Relative MPA (40% MET_max_^d^)	Relative VPA (60% MET_max_)
Multiple regression	107.7	133.9	116.2	130.9
Mixed model	108.2	134.5	117.3	131.5
ROC^e^ curve	104.3	140.0	118.5	127.3

^a^MPA: moderate-intensity physical activity.

^b^METs: metabolic equivalents.

^c^VPA: vigorous-intensity physical activity.

^d^MET_max_: estimated VO_2max_ divided by 3.5 mL/kg/min.

^e^ROC: receiver operating characteristic.

### Mixed Effects Modeling

**Figure 1:**
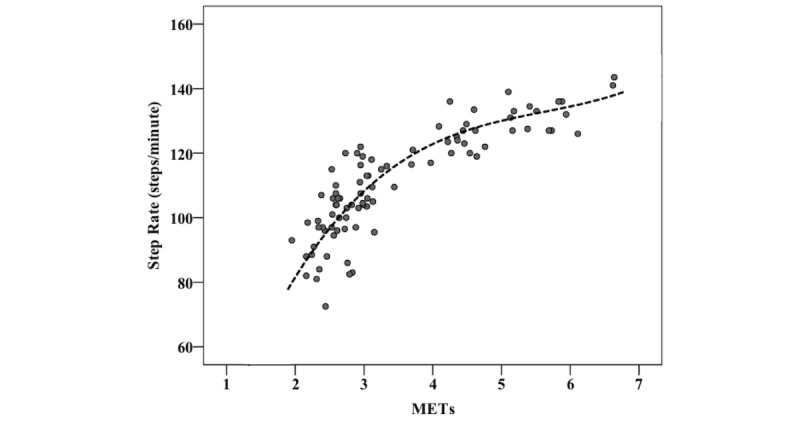
Scatter plot demonstrating the relationship between metabolic equivalents (METs) and step rate. The reference line is the mixed effects model using the sample’s median value for body mass index.

Similar to previous research [7], intercepts were allowed to vary among participants (ie, random intercept modeling was used). Regardless of intensity, the mixed effects model yielded similar step rates compared to the multiple regression model when BMI was used as a predictor (see Table 3). Figure 1 demonstrates the curvilinear relationship between step rate and metabolic activity with the mixed effects model as the reference line (median BMI 26.2 kg/m^2^). The influence of BMI on the relationship between step rate and metabolic activity is further presented in Table 4. Due the interaction effect between BMI and METs, a greater BMI was associated with faster step rates at 3 METs but with slower step rates at higher METs (ie, 5 METs and 6 METs). Likewise, the positive BMI coefficient (2.111×BMI) and the negative BMI*MET interaction (–0.549×BMI×METs) cancel out at 4 METs, resulting in no influence of BMI on step-rate thresholds at this intensity (see Table 4).

### Receiver Operating Characteristic Curves

Data used for absolute MVPA and relative MVPA calculations were dependent upon the average MET values for the respective walking stages. The absolute MPA (3 METs) ROC curve was generated based on stages 1 to 3 (mean ranging from 2.4 to 3.1 METs). The optimal step rate was 104.3 spm, with 83.3% correctly classified as achieving MPA and 71.1% correctly classified as not achieving MPA (AUC 0.791 [95% CI 0.658-0.923], SE 0.07, *P*=.002). The relative MPA (3.54 METs) ROC curve was generated based on stages 2 to 4 (mean ranging from 2.7 to 4.4 METs). The optimal step rate was 118.5 spm, with 83.3% correctly classified as achieving MPA and 86.8% correctly classified as not achieving MPA (AUC 0.939 [95% CI 0.878-0.999], SE 0.03, *P*<.001).

**Table 4:** The relationship between step rate and metabolic activity when adjusted for body mass index.

BMI^a^ (kg/m^2^)	Step rate (steps per minute)			
	3 METs^b^	4 METs	5 METs	6 METs
20	105	123	133	141
22	106	123	132	139
24	107	123	131	137
26	108	123	130	135
28	109	123	129	132
30	110	123	128	130
32	111	123	127	128

^a^BMI: body mass index (kg/m^2^).

^b^METs: metabolic equivalents.

For both relative and absolute VPA (5.31 METs and 6.00 METs), the ROC curve was generated based on stages 4 and 5 (mean ranging from 4.4 to 5.7 METs). The optimal step rate for relative VPA was 127.3 spm with 75.0% correctly classified as achieving VPA and 70.0% correctly classified as not achieving VPA (AUC 0.804 [95% CI 0.655-0.953], SE 0.004, *P*=.004). The absolute VPA ROC curve corresponded to a step rate of 140.0 spm with a sensitivity of 66.7% and a specificity of 100% but did not reach statistical significance (AUC 0.793 [95% CI 0.453-1.000], SE 0.17, *P*=.10).

## Discussion

### Principal Findings

The primary purpose of this study was to determine the step-rate thresholds associated with MPA and VPA in both absolute and relative terms for older adults. The most accurate model included BMI but not height or leg length as predictor variables in this diverse sample of older adults. In absolute terms, MPA (ie, 3 METs) and VPA (ie, 6 METs) corresponded to approximately 110 spm and approximately 135 spm, respectively. Unlike the previously established 100 spm required to achieve MPA in adults, the results of this study demonstrate that older adults need to walk faster to achieve the same intensity; however, this relationship is altered by BMI status. The sample’s relative MPA (ie, 3.5 METs) and relative VPA (ie, 5.3 METs) equated to approximately 117 spm and approximately 132 spm, respectively.

Recent reviews [19,20] discussing the cadence required to achieve MPA and VPA demonstrate the need for more evidence regarding the recommended walking intensity in older populations. Our observed results highlight the importance of considering participants’ physical characteristics when prescribing cadences in older persons. For MPA in absolute terms, older adults who have greater BMI need to take slightly more steps than those with a lower BMI (approximately 5 spm difference between 20 kg/m^2^ and 30 kg/m^2^). However, this relationship is reversed and magnified at higher MET values (eg, 5 to 6 METs) with older individuals who have higher BMIs needing to take fewer steps per minute to equate to the same intensity as individuals with lower BMIs (approximately 11 spm difference between 20 kg/m^2^ and 30 kg/m^2^). The observation that BMI influences MPA step-rate thresholds has been previously observed in young adults [9], in which they also observed negative interaction effects between BMI and METs (BMI*METs*–0.52) in their proposed model (*R*^2^=0.68). Contrary to their model, this study’s proposed equation (*R*^2^=0.77) was not improved by adding leg length or height as predictor variables, suggesting that BMI impacts the metabolic requirements of walking more than leg length and height in elderly individuals.

The existing studies in older persons have demonstrated that the relationship between cadence and MPA is influenced by either height [12] or body weight [11]. Of relevance, BMI and body weight were not explored as predictor variables by Peacock et al [11]. One limitation of the previous literature is that they modeled the relationship between step cadence and metabolic activity as linear, whereas we clearly demonstrate that it is curvilinear (see Figure 1), which would alter step rate predictions. Additionally, the model proposed by Serrano et al [12] uses body weight and self-selected walking cadence to predict MPA in relative terms; using self-selected cadence as a predictor variable decreases the practicality of their model in comparison to using height or BMI. The model proposed by Peacock et al [11] uses step rate as a predictor variable and METs as the outcome variable, requiring a back calculation of the required cadence for a given MET value. Consistent with some [9,24], but not all [11,21] previous research, we used step rate as the outcome variable because it does not require a back calculation of MET values making it is much easier to use by health care and exercise professionals in calculating their patients’ MVPA step-rate thresholds. As well, both methods produce almost identical values. We do acknowledge that metabolic intensity is a function of stepping cadence but we opted for an equation that is simpler, performs just as well, and is easier to use.

A systematic review by Slaght and colleagues [19] calls for more evidence regarding the prescription of walking cadence as a means of increasing physical activity in practical settings. Whether older adults achieve MPA at higher cadences than their younger counterparts (eg, 100 spm) is controversial in the literature; however, our results demonstrate that 3 METs is reached at 105 to 110 spm in older adults, depending on BMI status. Certainly, there is merit to the heuristic public health recommendation of 3000 steps in 30 minutes (ie, 100 spm). However, individualizing step-rate thresholds would minimize the potential error associated with not considering BMI. This is of particular importance when recommending cadences that equate to higher intensity physical activity as VPA step-based thresholds are more affected by BMI status (128 to 141 spm between 20 and 30 kg/m^2^; see Table 4). To the authors’ knowledge, this is the first study to evaluate VPA step-rate thresholds in older adults. Given the practicality of the 3000 steps in 30 minutes message for MPA, providers may consider recommending 4000 steps in 30 minutes (approximately 133 spm) as a general message to help their patients achieve VPA. However, the study’s reference equation is provided so that providers can individually tailor step rates when deemed appropriate (ie, a physical activity monitor study), based on a patient’s personal level of fitness and calculated moderate and vigorous MET targets. Of importance, our population is representative of a typical older Canadian adult with an average VO_2max_ of fair to good (ie, approximately 7 to 9 METs) [15,33], self-reported MVPA of approximately 30 minutes per day (or 210 minutes per week) [34], and a BMI in the overweight category (eg, average Canadian approximately 28 kg/m^2^) [35], which highlights the generalizability of this study’s results.

### Limitations

This study may be limited by our laboratory evaluations across a defined set of walking speed in that the proposed models are limited to step rates between approximately 90 to 135 spm. Although a broad range of walking conditions were used, more stages in the vigorous zone would have improved our predictions of VPA cadences. However, to our knowledge, this is the first study to investigate VPA step-rate thresholds in older populations. Furthermore, the findings of our study may be limited to BMI status’ as the range of BMIs in this sample was 20.6 to 31.2 kg/m^2^ and there were only 4 participants in the obese class 1 (30.0 to 34.9 kg/m^2^) category. However, the overall trend of lower VPA step-rate thresholds as BMI increases would likely not change if a greater proportion of obese persons were studied. Regardless, future research incorporating older adults of a broader range of BMI status’ (ie, obese class 2, obese class 3) are warranted. There may be differences in applicability to free-living conditions, although walking on a treadmill is kinetically and kinematically equivalent to walking over ground in healthy subjects [36]. As well, our sample size may be considered a limitation, but it is reflective of similar studies investigating MVPA step-rate thresholds [8,9,11,22,23], and participants completed up to 5 walking stages that resulted in multiple data points per subject. We do acknowledge that a larger sample size would likely corroborate the findings of this study and anticipate that the results from the forthcoming Cadence-Adults study will further strengthen recommendations for MVPA step-rate thresholds in young and older adults [20]. Lastly, the use of a submaximal assessment of aerobic fitness may be considered a limitation; however, the single stage treadmill protocol is a valid indicator of aerobic fitness [27] and the most practical for exercise professionals to adopt given their limited time allotted to assess patient fitness and provide an exercise program. Future research should evaluate the effectiveness of pedometer-based goals (ie, 3000 or 4000 steps in 30 minutes) in helping more of the older adult population achieve the national physical activity guidelines and reduce their risk of chronic disease.

### Conclusion

Walking is the most common form of leisure-time physical activity among older adults but the cadences required to achieve MPA and VPA in this population are understudied. This study provides evidence that older adults achieve absolute MPA and VPA at approximately 110 spm and approximately 135 spm, respectively, which is higher than the public health recommendation of 100 spm required to reach MPA previously reported. Further, anthropometric factors such as BMI significantly influence the curvilinear relationship between step rate and intensity-related physical activity. The findings of this study support that health care professionals and researchers should individualize MVPA step-rate thresholds based on their elderly patients’ body sizes and, when possible, use relative MPA and VPA values.

## Abbreviations

AIC: Akaike information criterion
AUC: area under the curve
BMI: body mass index
CSEP: Canadian Society for Exercise Physiology
MET: metabolic equivalent
METmax: relative estimated VO_2max_ divided by 3.5 mL/kg/min
MPA: moderate-intensity physical activity
MVPA: moderate-to-vigorous physical activity
PAR-Q+: Physical Activity Readiness Questionnaire Plus
PASB-Q: Physical Activity and Sedentary Behavior Questionnaire
ROC: receiver operating characteristic
RPE: rating of perceived exertion
spm: steps per minute
VO_2_: volume rate of oxygen consumed
VO_2max_: maximal aerobic fitness
VPA: vigorous-intensity physical activity
